# Supplementary figures and images for: Progression of herpesvirus infection remodels mitochondrial organization and metabolism
Source: PLoS Pathog. 2024 Apr 15;20(4):e1011829. doi: 10.1371/journal.ppat.1011829 (PMC11045090; doi:10.1371/journal.ppat.1011829)

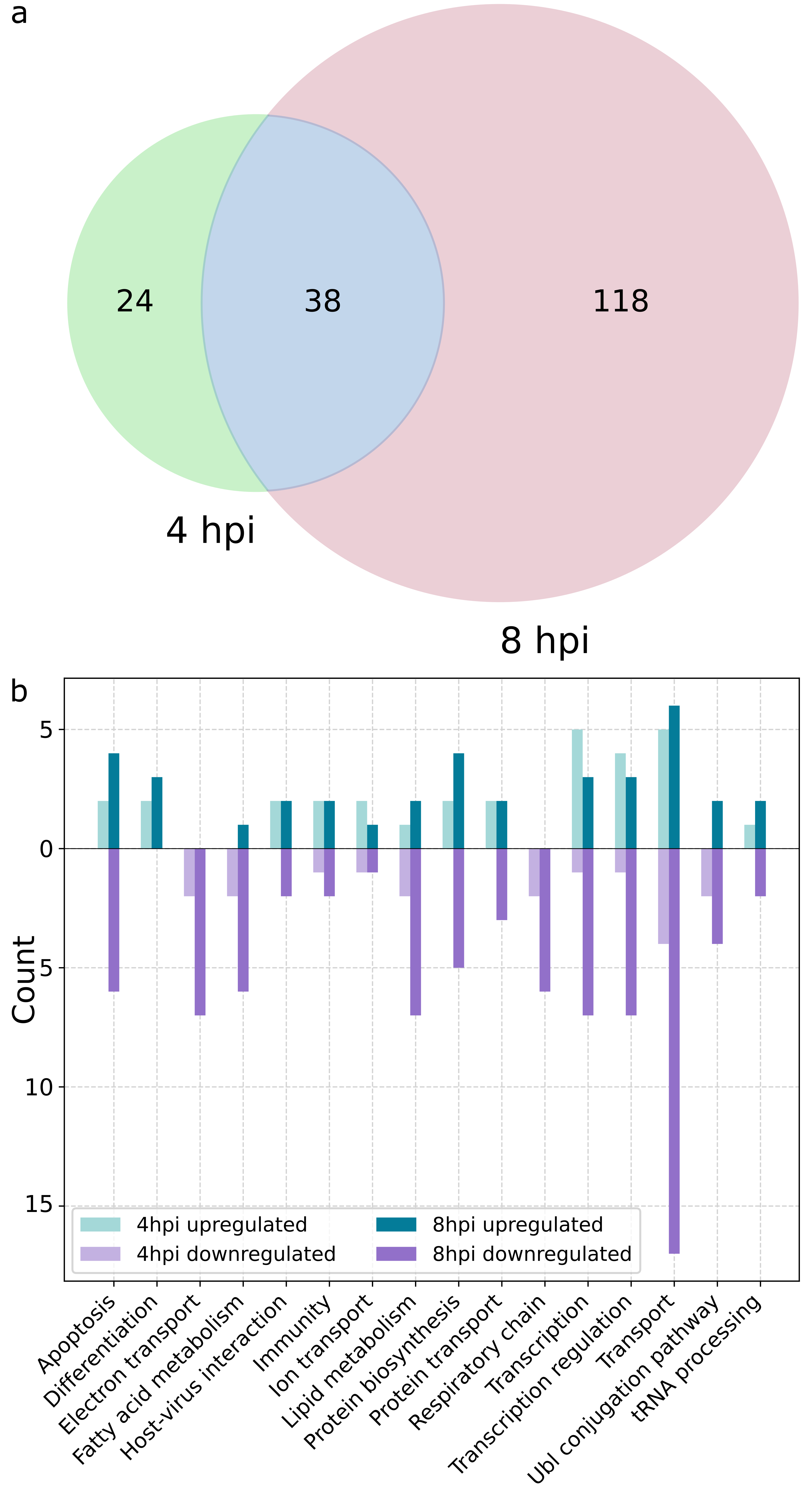

Supplement: S1 Fig — (a) A Venn diagram of the GRO-Seq dataset showing gene regulation of mitochondrial protein-associated gene transcription in infected Vero cells. The number of regulated proteins at 4 (green) and 8 hpi (pink), or at both time points (blue) is shown. The size of the circle is proportional to the number of regulated proteins. (b) The upregulation and downregulation of mitochondrial genes at 4 and 8 hpi clustered according to their predicted functional protein-protein interactions. GO terms of their functional classes of major biological processes detected in infection are shown. The horizontal line at value 0 represents gene transcription in noninfected cells, and positive and negative values represent the upregulation and downregulation of the gene transcription. (TIF) [file ppat.1011829.s001.tif]

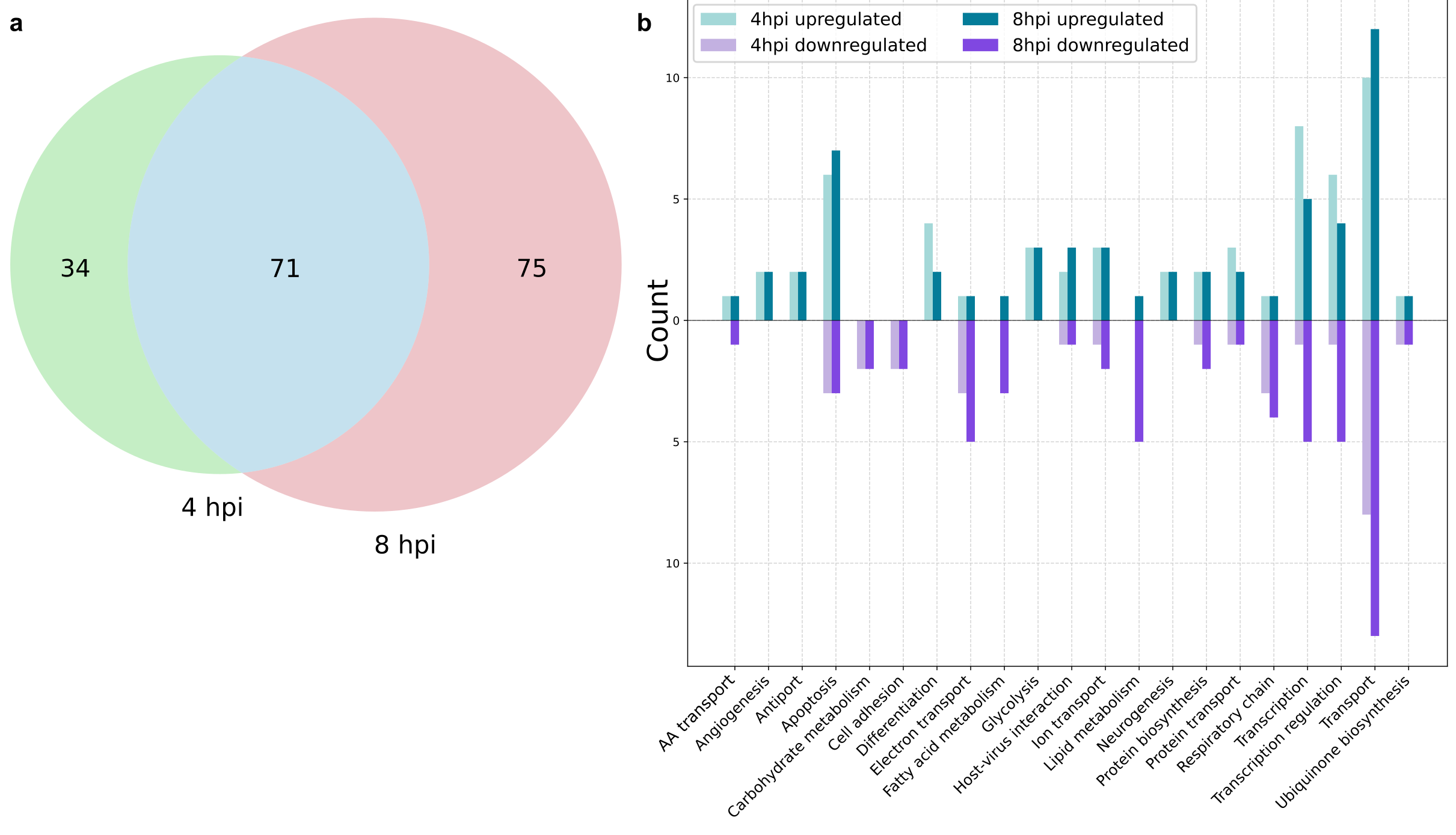

Supplement: S2 Fig — (a) A Venn comparison of GRO-Seq dataset showing gene regulation of mitochondrial protein-ssociated gene expression in infected Hela cells at 4 and 8 hpi. The number of regulated proteins at 4 (green) and 8 hpi (pink), or at both time points (blue) is shown. The size of the circle is proportional to the number of regulated proteins. (b) The upregulation and downregulation of mitochondrial genes at 4 and 8 hpi clustered according to their predicted functional biological process during infection. The horizontal line at value 0 represents gene expression in noninfected cells, and positive and negative values represent the upregulation and downregulation of gene expression, respectively. (TIF) [file ppat.1011829.s002.tif]

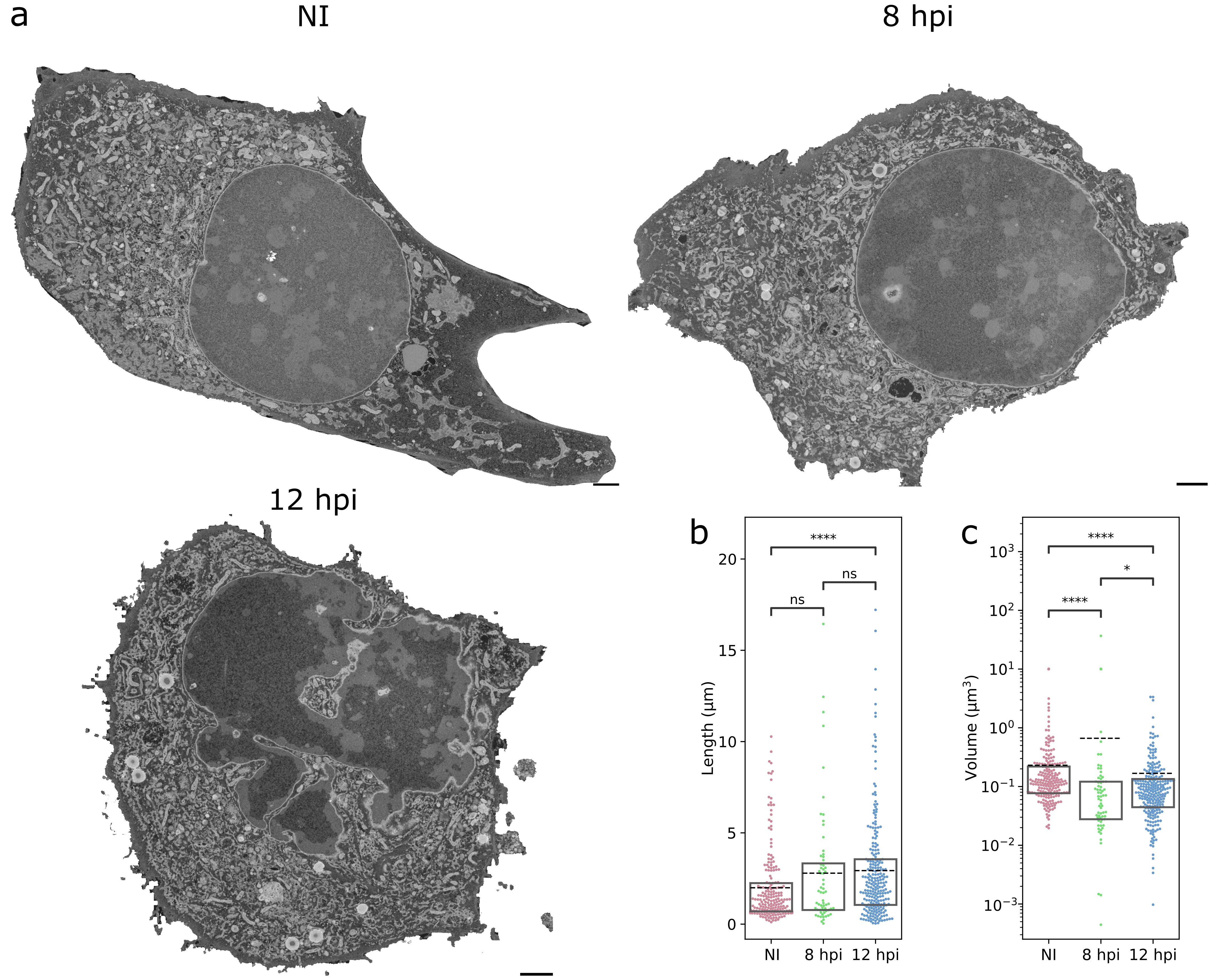

Supplement: S3 Fig — (a) Serial block face scanning electron microscopy (SBF-SEM) images of noninfected and infected MEF cells at 8 and 12 hpi. Scale bars: 2 μm. (b) Quantitative analysis of mitochondrial length (nmito = 202, 64, and 244 for NI, 8, and 12 hpi, respectively). The box plots show the mean (dashed line) and the interquartile range. Statistical significance was determined using the Student’s t-test. The significance values are denoted as **** (p<0.0001), *(p<0.05), or ns (not significant). (TIF) [file ppat.1011829.s003.tif]

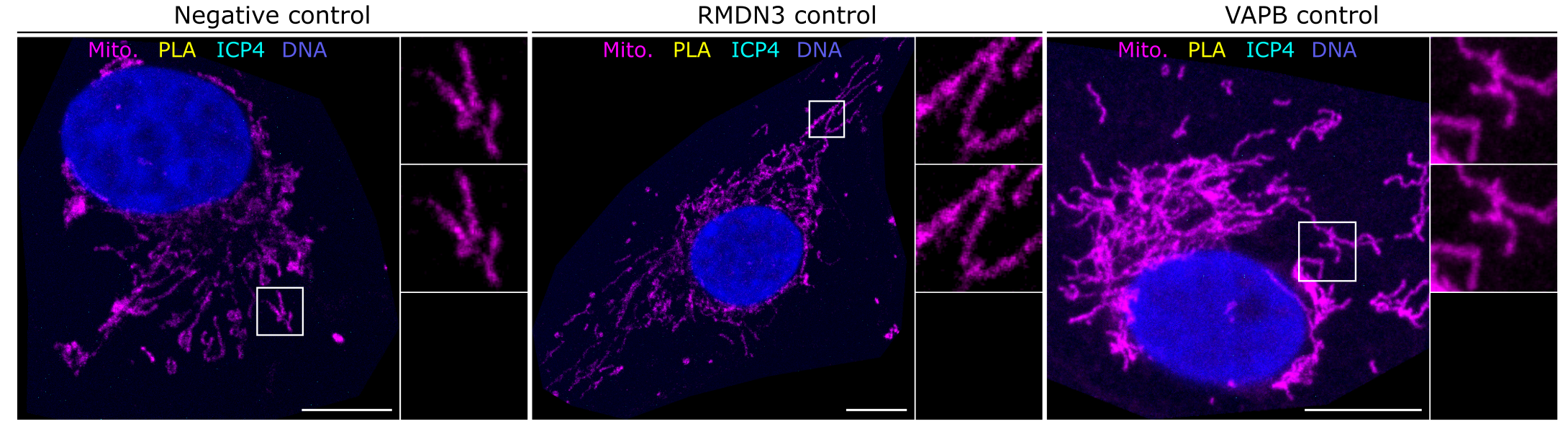

Supplement: S4 Fig — Representative images of the PLA technical controls showing the PLA signals in noninfected cells either without the PLA probes (left), without the VAPB antibody (middle), or without the RMDN3 antibody (right). The PLA signals between VAPB and RMDN3 are visualized in yellow. Mitochondria are labeled with MitoTracker (magenta), the nucleus with DAPI (blue), and EYFP-ICP4 is used as the viral marker (cyan). The marked areas are magnified on the right side of each image, showing the PLA signal together with the mitotracker, only the mitotracker, and only the PLA signal, respectively. Scale bars, 10 μm. (TIF) [file ppat.1011829.s004.tif]

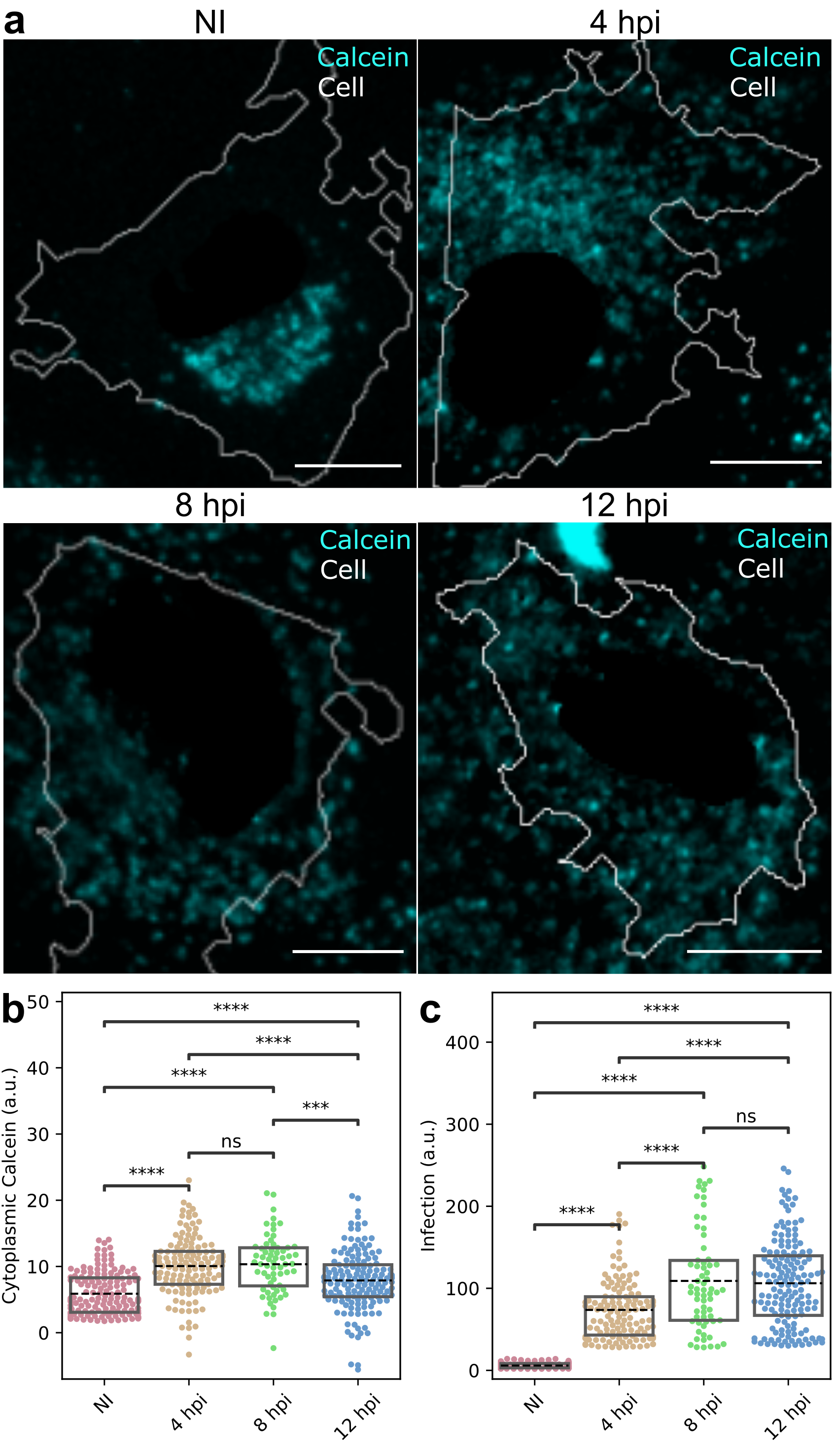

Supplement: S5 Fig — The state of mPTP was assessed by cellular loading of fluorescent Calcein-AM and Co2+ quencher. (a) Representative cells showing Calcein (cyan) distribution in the cytosol of noninfected and infected MEF cells at 4, 8, and 12 hpi. The plasma membrane localization is shown (cell, grey line). (b) The fluorescent intensity of cytoplasmic Calcein and nuclear viral replication compartment marker, ICP4, in noninfected and infected cells at 4, 8, and 12 hpi (n = 150, 134, 65, and 152, respectively). Statistical significance was determined using the Student´s t-test. The significance values are denoted as **** (p<0.0001), *** (p<0.001), or ns (not significant). (TIF) [file ppat.1011829.s005.tif]
