# Supplementary material for: Progression of herpesvirus infection remodels mitochondrial organization and metabolism
Source: PLoS Pathog. 2024 Apr 15;20(4):e1011829. doi: 10.1371/journal.ppat.1011829 (PMC11045090; doi:10.1371/journal.ppat.1011829)
Supplement: Attachment — Submitted filename: Revision letter.pdf [file ppat.1011829.s015.pdf]

PLOS Pathogens
Editors
Eain A Murphy, Ph.D.
Patrick Hearing
Kasturi Haldar
Michael Malim

March 1st 2024

Dear Editors,

Please find enclosed the revised manuscript (PPATHOGENS-D-23-02006) "Progression of herpesvirus infection remodels mitochondrial organization and metabolism" by Simon Leclerc et al.

We greatly appreciate the opportunity to revise our manuscript. As outlined in our response letter, we have now addressed all the points raised by the reviewers. Based on their suggestions, appropriate changes have been introduced to the manuscript. We trust we have been able to address their concerns and, hence, improve our work.

We hope our manuscript is now suitable for publication in PLOS Pathogens.

Yours sincerely,

Maija Vihinen-Ranta et al.
Department of Biological and Environmental Science
University of Jyväskylä, Finland
Phone: +358 400 248 118
Email: maija.vihinen-ranta@jyu.fi

Our responses to the reviewers' comments

Part II – Major Issues: Key Experiments Required for Acceptance

Reviewer #1:

It is puzzling that the authors selected the cell types they did for these studies, and I feel that these choices undermine the impact of the report.

MEFs and microscopy: Understandably, MEFs are amenable to microscopic approaches given their morphology. However, MEFs aren't naturally infected with HSV-1, and their true value as an infection model (interrogation of gene deletions on infection) is not leveraged in this study. It is difficult to understand why in the very least a human cell line such as U2OS, hTERT-immortalized normal dermal fibroblasts, or better, a cell model for human neurons such as SK-N-SH, or SH-SY5Y, or best, iPSC-derived human neurons was not used for the mitochondrial morphology/structure analyses. Since mitochondrial organization and ultrastructure vary widely, using a more appropriate cell type would have elevated the impact of this study substantially. At the end of the study the reader interested in mitochondrial dynamics during a more natural HSV-1 infection is left asking "what about in human neurons?", a question that will have to be answered by another study. Since the elegant microscopy approaches used here are highly specialized, requiring advanced technical expertise and beamline access, recapitulating these studies in neurons is not trivial for the majority of the interested readership.

We are deeply indebted to all Reviewer #1 for his/her constructive comments and invaluable help in improving our manuscript.

The morphology of mouse embryonic fibroblasts (MEF) is indeed optimal for our imaging techniques. Fibroblasts have been used in other published papers with HSV-1 (Tiwari et al. 2006). We have used MEF cells in several so far unpublished projects in addition to this manuscript. Besides our experiments, earlier growth curve analyses of the Joel Baines group have verified the HSV-1's infectivity and ability to produce infectious viruses in MEF cells (Mou et al. 2008).

We truly agree that studies with human neuronal cells (e.g. SH-SY5Y) would be an excellent next step in our studies.

Ref.

Tiwari V, Clement C, Xu D, Valyi-Nagy T, Yue BY, Liu J, Shukla D. Role for 3-O-sulfated heparan sulfate as the receptor for herpes simplex virus type 1 entry into primary human corneal fibroblasts. J Virol. 2006 Sep;80(18):8970-80. doi: 10.1128/JVI.00296-06.

Mou F, Wills EG, Park R, Baines JD. Effects of lamin A/C, lamin B1, and viral US3 kinase activity on viral infectivity, virion egress, and the targeting of herpes simplex virus U(L)34-encoded protein to the inner nuclear membrane. J Virol. 2008 Aug;82(16):8094-104. doi: 10.1128/JVI.00874-08.

Veros and transcriptomics, PLA: What is the rationale behind using Vero cells? The GRO-seq analysis of mitochondrial gene expression was carried out using HSV-1-infected Vero cells. Veros are instrumental in growing viruses owing in part to their

lack of interferon production (Emeny and Morgan, J Gen. Virol., 43(1):247, 1979). Given this, using Veros for gene expression profiling at 4 and 8hpi, during which interferon responses are at their maxima in interferon-producing cells, will miss a plethora of interferon stimulated genes, many of which influence mitochondrial-associated innate immunity, activity, and shape. Mitochondrial antiviral defenses are mentioned regarding Figure 1A and B (Results, page 5, and again on page 8 before imaging section), but the transcriptional response shown lacks a large complement of ISGs.
The TREx/PLA and transcriptomic studies were also performed using Vero cells. Since the PLA is an extension of the work on mitochondrial-ER contact sites in MEFs, why was the cell type switched? What is the extent to which the MEF studies can be augmented by intramolecular interaction studies in a heterotypic cell line?

Thank you for this highly relevant point. Based on this, we analyzed the host transcriptome of interferon-producing Hela cells at 4 and 8 hpi. Our GRO-seq data revealed a relatively similar pattern of the host cell transcription in both Vero and HeLa cells, however, with some cell line-specific variations (a new supplementary Fig. 2). Interestingly, analysis of upregulation and downregulation of mitochondrial genes at higher levels in the GO hierarchical structure (level 0) showed regulation of immunity in MEF but not in HeLa cells. We have modified the text (Results page 5, lines 140-142) and added Fig. S2.

The PLA interaction analysis of VAPB and RMDN3 (PTPIP51) proteins are detected by VAPB MAb, paired with Rabbit RMDN3 Ab. The detection is possible in African green monkey kidney cells (Vero) but not in mouse embryonic fibroblast cells *(*MEFs) (background labeling). Unfortunately, antibodies functional in the MEF cells were not available.

Expanding these studies from our model cell lines (MEF, Vero) to heterotypic cell lines would be an interesting project.

Reviewer #2: 1. The rationale for selecting the specific time points of 4, 8, and 12 hours post-infection (hpi) needs clarification. In some experiments, only 2 out of 3 time points were selected. It is recommended to supplement the missing time point data or should explain the reason.

Our experiments aim to compare events in early infection (4 hpi) with late infection (8 and/or 12 hpi). At 4 hpi, the viral replication compartment emerges and starts to grow. Later, at 8 and 12 hpi, the replication compartment is enlarged, viral late proteins are expressed, and assembled capsids egress the nucleus. At this stage, the cell is still actively producing ATP. Remodification of cellular structures and organelles takes place during late stages (Scherer et al. 2021). Even in late stages, the cellular mitochondrial ATP machinery remains active until 18 hpi, when ATP production declines, mitochondria become fragmented, and its DNA is released into the cytosol (Murata et al. 2000; Saffran et al. 2007; Berry et al. 2021).

The previous studies described above suggest that there is a clear change in cellular and, most likely, in mitochondrial structure and function when infection proceeds to late infection (8, 12 hpi). Our GRO-seq, cryo SXT microscopy (in capillaries, Lawrence Berkeley National Laboratory, CA), PLA, and Seahorse experiments included time points 4 and 8 hpi and noninfected control cells. Moreover, cryo SXT (on grids, ALBA light Source, Barcelona,

Spain) SBF-SEM contain noninfected and late infection time points (8,12 hpi) to highlight events in the late infection. We admit that it would have been good to include also 4 hpi in these experiments. However, due to practicalities (costs, access to facilities, etc.), the repetition of the experiments was too challenging. The FIB-SEM imaging and analysis requiring laborious manual segmentation was performed only in noninfected and infected cells at 8 hpi.

Refs:

Scherer KM, Manton JD, Soh TK, Mascheroni L, Connor V, Crump CM, Kaminski CF. A fluorescent reporter system enables spatiotemporal analysis of host cell modification during herpes simplex virus-1 replication. J Biol Chem. 2021 Jan-Jun; 296:100236. doi: 10.1074/jbc.RA120.016571.

Murata, T. et al. Mitochondrial distribution and function in herpes simplex virus-infected cells. J. Gen. Virol. **81**, 401–406 (2000).

Saffran, H. A., Pare, J. M., Corcoran, J. A., Weller, S. K. & Smiley, J. R. Herpes simplex virus eliminates host mitochondrial DNA. EMBO Rep. **8**, 188–193 (2007).

Berry, N. *et al.* Herpes Simplex Virus Type 1 Infection Disturbs the Mitochondrial Network, Leading to Type I Interferon Production through the RNA Polymerase III/RIG-I Pathway. *mBio* **12**, e0255721 (2021).

2. In Fig 2j, there are only 4 cells at 8 hpi, significantly fewer than NI and 12 hpi groups. Is there a factor of artificial exclusion? What impact might this have on the statistical results?

The number of cells visualized at 8 hpi in this dataset was lower. The combination of access to the synchrotron, grid quality, and acquisition time made us decide to focus more on the noninfected and 12 hpi conditions. However, the number of mitochondria analyzed is still high even at 8 hpi (hundreds of them analyzed). Moreover, cell-to-cell variation of infected cells was low. To verify this, we calculated the cellular mean and standard deviation values, and the standard deviation was less than 10% of the mean. For example, the mitochondria roughness was 41.41 um (+- 3.84), or the distance to the nucleus 9.72 um (+-0.58).

3. In this work, the Mitochondrial permeability transition pore (mPTP) of Ca2+ entering mitochondria has not been given attention, especially the open state of this channel complex, would the author add this experiments. In addition, the effect of HSV-1 virus protein on mPTP has been reported (PMID: 34234233, 34234233), and it is recommended to supplement the relevant discussion.

This was a very good addition to the manuscript. The measurement of the regulation of mitochondrial permeability transition pores (mPTP) has been added to the manuscript (Results page 12, lines 303-310, Supplementary Figure 5 and methods; Discussion page 16, lines 398-403).

Appreciating the Reviewer’s suggestion, we have added HSV-1 UL16 interaction with mitochondrial permeability transport channel component, adenine nucleotide transporter 2 (ANT2), and its activating effect on ATP generation into the Discussion (page 16, lines 398-403).

4. What is the basis for selecting VAPB and RNDM3 as representative proteins in the study of interactions between mitochondria and their inner membrane proteins?

ER-mitochondria linkers VAPB and RMDN3 (PTPIP51) have been recently used in elegant studies of ER-mitochondria contact sites in HCMV, HSV-1, Influenza A, and beta-coronavirus HCoV-OC43 infections. In addition to their wide use in ER-mitochondria contact site studies, this assured us to use these proteins.

Ref.
Cook, K. C., Tsopurashvili, E., Needham, J. M., Thompson, S. R. & Cristea, I. M. Restructured membrane contacts rewire organelles for human cytomegalovirus infection. *Nat. Commun.* **13**, 4720 (2022).

5. Electron microscopy and 3D reconstruction are a major methodological innovation in this work, providing direct evidence of the impact of HSV-1 on host cell mitochondria. However, could the author provide direct evidence of the interaction between HSV-1 and mitochondria through SEM supplementation?

We assume that the interaction between HSV-1 and mitochondria would be mediated via viral proteins. It would be interesting to study the distribution and interactions of viral proteins located in the mitochondria, including UL7, UL16, US3, UL12.5, and their mutants. The techniques utilized could include 3D confocal microscopy, expansion microscopy, and super-resolution fluorescent microscopy. In addition, EM techniques such as pre-embedding nanogold allowing antibody labeling of the proteins and correlative light and electron microscopy (CLEM) could be used. Using 3D volume EM applications (SBF-SEM, FIB-SEM) to detect viral proteins could be challenging, but not impossible.

6. The imaging data related to mitochondria in the article is manually segmented; therefore, the basis for image segmentation should be described in the methods section. Furthermore, it seems that mitochondria cannot be visually distinguished from the image in Fig 2a, f.

The Methods section in the Supplementary material now contains the updated version of the various imaging data segmentation processes. We have also added Supplementary Table 4, summarizing the segmentation protocols used in various imaging techniques. Shortly, in automatically reconstructed SXT projection images of suspended cells in capillaries, mitochondria were segmented manually, using commercial software Amira-Avizo (Thermo Fisher Scientific) based on the morphology or LACs (Fig. 2). From the aligned stacks of Cryo-SXT of adherent cells on grids, the mitochondria were segmented using Biomedisa (https://biomedisa.info/) and analyzed by using X-ray absorption threshold analysis (Fig. 2). The manual segmentation was used in the segmentation of mitochondria and ER from SBF-SEM data (Fig. 3). The mitochondrial cristae were semiautomatically segmented from FIB-SEM using Microscopy Image Browser (MIB) segmentation tools (Fig. 5).

We agree that SXT images look different from EM images, for example. Cryo-SXT imaging is based on the absorption of X-rays in carbon-rich materials such as biological membranes, including membraneous mitochondria. Mitochondria can be identified from SXT tomograms by combining their linear absorption coefficient values and general morphology (Loconte, White, 2022).

Ref. Loconte V, White KL. The use of soft X-ray tomography to explore mitochondrial structure and function. Mol Metab. 2022 Mar;57:101421. doi: 10.1016/j.molmet.2021.101421.

Reviewer #3: Although the authors have presented original approaches with clear and demonstrative high-resolution images, the work presented in this article does not represent a major advance in the field. Indeed, there is no data specifying the potential role of HSV-1 during infection. No experiments specifying the impact of the virus on the perturbation of the mitochondrial network were presented. Important controls are missing, such as infection efficiency and viral replication. There is no imaging of colocalization between viral and mitochondrial proteins. These data could ultimately be presented with any pathogen/virus as we do not observe virus specificity during disruption of the mitochondrial network. The seahorse experiments on mitochondrial metabolism have already been reported by other groups, as well as images of mitochondrial remodeling.

We are thankful to Reviewer 3 for pointing out these important issues.

The HSV-1 infection-induced changes in the mitochondrial network have been presented earlier. However, we think that our multimodal integrated state-of-the-art techniques combined with quantitative data analysis provide morphological evidence and draw a more comprehensive picture of time-dependent changes in mitochondria as HSV-1 infection proceeds from early to late infection. Specifically, our analysis revealed an extensive growth in the number, volume, and density of ER-mitochondria contact sites at late infection simultaneously with an increase in mitochondrial membrane roughness and mitochondrial Ca^{2+} content. The increased availability and clustering of contact sites most likely result in enhanced Ca^{2+} flux from the ER to mitochondria. This most likely explains the reactivation of respiration at late infection and possibly also the perinuclear localization of mitochondria. These events are supported by our results showing that the expression of the mitochondrial pore-forming subunit of calcium voltage-dependent calcium channel subunit (CACNA1B) is upregulated in infection. Previous studies have demonstrated the progression of infection in Vero and MEF cells (e.g. Mou et al. 2008).

Ref.
Mou F, Wills EG, Park R, Baines JD. Effects of lamin A/C, lamin B1, and viral US3 kinase activity on viral infectivity, virion egress, and the targeting of herpes simplex virus U(L)34-encoded protein to the inner nuclear membrane. J Virol. 2008 Aug;82(16):8094-104. doi: 10.1128/JVI.00874-08.

It would be interesting to take this work further by highlighting the impact of the virus. Which viral proteins target mitochondria? Are there interactions between viral proteins and mitochondrial proteins or ER proteins? Is this phenomenon of mitochondrial disruption directly influenced by the presence of HSV-1 or is it an indirect phenomenon? This is interesting work, but highly incomplete.

Studying the distribution and interactions of HSV-1 proteins in the mitochondria would be very exciting, especially including viral proteins UL7, UL16, US3, and UL12.5 in these studies. The role of specific viral proteins in mitochondrial disruption could be monitored by using mutant viral proteins. The interaction of viral proteins with cellular and mitochondrial proteins could be studied by protein-protein interaction analyses, e.g., bioID. However, these extensive studies would be an interesting next step in our studies.

Part III – Minor Issues: Editorial and Data Presentation Modifications
Please use this section for editorial suggestions as well as relatively minor modifications of existing data that would enhance clarity.

Reviewer #1: 1. General: The broad readership of PLOS Pathogens would very likely benefit from a table (supplemental) summarizing the different microscopy modalities used herein, ie: where that approach excels; what its basis is; what its benefits/drawbacks are (resolution vs. volumetric rendering), etc. Such a table would endow the reader with appreciation for the technical possibilities in ultrastructural biology (given beamline access), clarify the approach rationale, and make the figures easier to digest.

The table summarizing the description of microscopy modalities used here is attached to the manuscript as a supplementary Table 4.

2. Font size and figure resolution: The font size and/or figure resolution in several areas needs to be increased. Figure 1 A and B should be as large as allowable so that they are readable. The node labels in 1A are unreadable even upon zooming into the PDF.

Figure 1 has been modified, and the font size of Figs 1a and b has been increased.

In Figure 2, graphs C-J are very difficult to read, as are Figure 6 graphs B-G. Figure 6F would benefit also from even a modest enlargement.

Figures 2 and 6 have been modified and clarified, and the font size of quantitative box blot analyses has been increased.

The font should be increased in Supplemental Figures 1A and 2B and C.

The font size has been increased.

3. Page 3: Wording/clarity: "…complexes between mitochondria and the ER have been identified, including VAPB and PTPI51, respectively."

The sentence has been modified to form "The formation and maintenance of contact sites are mediated by tethering proteins, such as VAPB and PTPIP51" (Introduction, page 3, lines 94-95)

4. Page 5: Wording/interpretation: The last sentence of the introduction is overly general. State HSV-1, as different DNA viruses, likely affects mitochondria in ways other than described here.

Two last sentences of the introduction have been merged and reorganized to make a more specific statement (Introduction, page 5, lines 129-133).

5. Page 8: Clarity: Suggest shuffling the first 4 sentences of cryo-SXT below the "To create a comprehensive view…" sentence so that the rationale is introduced before the

method. Note: the use of a method table as suggested in point 1 above would help with this.

The sentences were relocated as suggested and the method table (Table 4) has been added.

6. Page 8: Clarity: One sentence explaining the use of suspended vs. grid-grown MEFs and what each adds to the data outcome would be useful.

The comparison of the SXT imaging in capillaries and on grids was added to the results (Page 8, lines 215-219). In addition, the new Table 4, shows the details of these techniques.

7. Figure 2: Clarity: Suggest a figure heading above A-C of "suspended MEFs" and over F-J of "grid-adhered MEFs".

Figure 2 was modified as suggested.

8. Page 9: Interpretation: Suggest "…distance between the ER and mitochondria is maintained owing to the protein-mediated…

This sentence has been remodeled.

9. Figure 4: Clarity: Suggest along the left of A a heading of "co-staining" and to the left of C a heading of "proximity ligation analysis", as this is a complex figure to unwrap. Other: In A, VAPB appears yellow, not green; in A there is no 12hpi timepoint as stated in the legend; in the legend it would be helpful to state that the replication compartments are nuclear; there is no red dashed line in B.

The Figure has been modified as suggested, and the figure legend has been corrected.

10. Page 10 and Figure 4: Interpretation: "In infected cells at 8 hpi VAPB was distributed heterogeneously and formed clusters along the mitochondria, in contrast to the more uniform distribution seen in noninfected cells (Fig. 4a). VAPB also moved further away from the mitochondria in infected cells when compared to noninfected cells (Fig. 4b)." In Figure 4A there is very little staining for both VABP and mitotracker. Is this due to the TREx process? To my eye it appears that there is more VAPB in uninfected than infected (not surprising given translational arrest). However, I don't see that distribution at 8hpi is "heterogeneous" or in "clusters" from this figure. Since the distance between mitochondria and VABP is significantly larger in Figure 4B, there must be a better example to show as 4A.

In the TREX process, samples are expanded in axial and lateral directions, increasing the sample thickness. This means that the staining gets more scattered around in 3D volume, and imaged slices have less concentrated staining (e.g., VAPB, mito tracker) than normal confocal slices. The infection-induced decrease in VAPB was added to the text, and a more representative image showing the heterogeneity and clustering was added to Fig. 4a.

11. Regarding Figure 4: Rigor: The negative controls for the PLA labeled with only one probe/sample should be provided, perhaps as a supplemental figure.

A new supplementary Figure 4 showing PLA control cells has been added.

12. Figure 6: Clarity: Parts C and D are switched in the figure legend. Regarding F, the mitotracker and rhod-2 AM appear weaker in the uninfected cells than in infected cells. However, in G the difference for 4hpi and uninfected is insignificant. Does the gain in F need to be adjusted.

"(**c**) ATP production, (**d**) proton leak" are now in proper order. We remodified Figure 6f by adding representative cells, which were in line with the quantitative data shown in Figure 6g.

13. Suppl. Figure 2: Clarity: The quality of the EM images should be improved. Also, lightening the cell hue will make it easier to see the organelle structures. Finally, the orange and blue outlines are not explained in the legend.

Unfortunately, the Supplementary pdf's resolution, pixels per inch (PPI), was low. The original SBF-SEM images are high resolution. We added contrast to the images. Finally, the unnecessary orange and blue outlines were removed.

14. Suppl. Figure legends: Clarity: For Movie 2, what are the yellow and green organelles? For Movie 3, it would be helpful to have a pseudocolor scale bar in the movie.

The figure legend for movie 2 has been modified, and information about the organelles- the nucleus (blue), mitochondria (light brown), lipid droplets (yellow), and multivesicular bodies (green) has been added. In addition, a pseudocolor scale bar was added to movie 3 and the figure legend.

Reviewer #2: 1. The descriptions of abbreviations such as ER (endoplasmic reticulum) and EM (electron microscopy) in the article are unclear.

The abbreviation of endoplasmic reticulum (ER) was added when first mentioned on page 3 (line 92) and electron microscopy (EM) on page 8 (line 209).

2. The fluorescence indicators (such as Mito/RMDN3/DAPI) in Fig 4 should be directly labeled on the fluorescence images for easier identification.

The fluorescence indicators were added to the Figure 4.

3. The size of Fig 6f is too small, and the details are not clear; it needs to be adjusted for better data presentation.

The Figure 6 f was enlarged to clarify the data.

Reviewer #3: (No Response)

PLOS authors have the option to publish the peer review history of their article (what does this mean?). If published, this will include your full peer review and any attached files.

If you choose "no", your identity will remain anonymous but your review may still be made public.

Do you want your identity to be public for this peer review? For information about this choice, including consent withdrawal, please see our

Reviewer #1: No

Reviewer #2: No

Reviewer #3: No
